# Estrogen administration enhances the adverse effects of cigarette smoking on the heart in cycling female mice

**DOI:** 10.1186/s13293-024-00667-3

**Published:** 2024-12-04

**Authors:** Emna Abidi, Reine Diab, Rana Zahreddine, Ghadir Amin, Abdullah Kaplan, George W. Booz, Fouad A. Zouein

**Affiliations:** 1 Department of Pharmacology and Toxicology, American University of Beirut Faculty of Medicine, American University of Beirut & Medical Center, Riad El-Solh, Beirut, 11072020 Lebanon; 2 The Cardiovascular, Renal, and Metabolic Diseases Research Center of Excellence, American University of Beirut Medical Center, Riad El-Solh, Beirut, Lebanon; 3 Department of Pharmacology and Toxicology, School of Medicine, University of Mississippi Medical Center, Jackson, MS USA; 4 Department of Signaling and Cardiovascular Pathophysiology, Institute Paris-Saclay for Therapeutic Innovation, Faculty of Pharmacy, University Paris Saclay, Orsay, France

**Keywords:** Cardiac remodeling, Tobacco use, Oxidative stress, Oral contraceptives, Inflammation, Cytokines

## Abstract

Smoking, particularly chronic smoking (CS), is a threat to global health, contributing to increased mortality and morbidity associated with cardiovascular disease (CVD). CS induces oxidative stress and endothelial dysfunction, which has a profound impact on cardiac structure and function. While the protective effects of estrogen, particularly 17β-estradiol (E2), on cardiovascular health are well-documented in premenopausal women, the interaction between estrogen and CS remains poorly understood. The aim of this study is to investigate the impact of chronic cigarette smoking on cardiac health in relation to ethinylestradiol (EE) oral contraceptive (OC) usage in premenopausal females. Female mice were exposed to chronic cigarette smoke and co-administered EE. Cardiac structural and functional parameters were assessed alongside inflammatory markers, oxidative stress indicators, and histological changes. Results revealed that the combination of EE and CS led to adverse cardiac remodeling characterized by increased left ventricular end-diastolic volume and elevated left ventricular mass. In addition, an inflammatory state was evident, marked by increased expression of IL-4, IL-1β, IL-13, IL-10, and PARP-1, as well as increased interstitial collagen deposition. These findings suggest a progression towards adverse cardiac remodeling resembling dilated cardiomyopathy. Furthermore, our observations highlight the complexity of the inflammatory response triggered by smoking, potentially exacerbated by estrogen supplementation. The main finding of this study is that the combination of CS and EE enhanced adverse cardiac remodeling, which was shown structurally, histologically, and biochemically.

## Highlights

- To assess the interaction between estrogen supplementation as seen with oral contraceptives and chronic smoking, female mice were exposed to cigarette smoke for 8 weeks and co-administered estrogen.
- The combination of estrogen and smoking led to adverse cardiac remodeling characterized by increased left ventricular end-diastolic volume and elevated left ventricular mass, suggesting a progression towards remodeling resembling dilated cardiomyopathy.
- In addition, an inflammatory state was evident, marked by increased expression of IL-4, IL-1β, IL-13, IL-10, and PARP-1, as well as increased interstitial collagen deposition.
- In contrast, during the 8-week timeframe, smoking by itself did not have structural or functional effects on the hearts of male mice or on female mice, regardless of the presence or absence of endogenous estrogen.
- Our findings highlight the complexity of the inflammatory response triggered by smoking as exacerbated by estrogen supplementation.

## Plain language summary

Chronic tobacco smoking contributes to increased mortality and morbidity and is associated with cardiovascular disease. Before menopause, the hearts of women are less affected by smoking due to the protective effects of the hormone estrogen. However, the interaction between estrogen and smoking remains poorly understood in premenopausal females, which was the focus of our study. Such would be the case with smoking woman on oral contraceptives. Female mice were exposed to chronic cigarette smoke for 8 weeks and co-administered estrogen. Heart structure and function were assessed, together with cardiac inflammation, oxidative stress, and histology. Our results revealed that the combination of estrogen and chronic smoking led to adverse cardiac remodeling that was characterized by increased volume of the relaxed left ventricle and elevated left ventricular mass. In addition, an inflammatory state was evident, marked by increased expression of several cytokines and heart fibrosis. These findings suggest a progression towards adverse cardiac remodeling resembling dilated cardiomyopathy. In contrast, during the 8-week timeframe, smoking by itself did not have structural or functional effects on the hearts of male mice or on female mice, regardless of the presence or absence of endogenous estrogen. Thus, our findings highlight the complexity of the inflammatory response triggered by smoking as exacerbated by estrogen supplementation. Further preclinical animal and clinical studies are needed to define the mechanisms behind these adverse effects.

## Introduction

Tobacco use and chronic smoking (CS) constitute a global epidemic among all age groups, causing serious health problems and a dramatic rise in deaths [1]. Tobacco smoke contains more than 4720 harmful compounds, such as polycyclic aromatic hydrocarbons, free radicals, and oxidative gases, which cause cells that line blood vessels to become swollen and inflamed. Indeed, CS is strongly associated with elevated oxidative stress status as evidenced by an increase in reactive oxygen species (ROS) production, LDL oxidation, attenuated levels of the cardioprotective HDL, higher levels of triglycerides, and subsequent endothelial dysfunction [2]. Moreover, CS has been demonstrated to be an independent risk factor accounting for 10% of cardiovascular diseases (CVDs), such as myocardial infarction, stroke, hypertension, coronary heart disease (CHD), atherosclerosis, and aortic aneurysm [3]. In fact, CS increases the risk of coronary artery disease (CAD) by 2- to fourfold with the CHD incidence rate in all age groups being highly attributable to smoking [4, 5].

CS may damage the heart through a direct effect on the myocardium resulting in smoking cardiomyopathy and an indirect effect by inducing other cardiovascular complications that in time damage and remodel the heart [6]. A source of oxidative stress that fuels the inflammatory response with CS is cellular and metabolic impairment; an excess in oxidants induces irreversible damage to cellular components, leading to altered cellular function or apoptosis [7]. CS also stimulates the recruitment of inflammatory cells leading to the generation of ROS, which contribute to protein oxidation and endothelial dysfunction. ROS production also triggers inflammation by effects on innate and adaptive immune cells, which in turn increase the secretion of pro-inflammatory cytokines [8]. ROS overproduction is characterized by the imbalance between antioxidant protection and ROS production [6, 9], leading to various alterations at the structural, molecular, cellular, and interstitial levels of the heart and clinically relevant changes in cardiac size, mass, geometry, and function [10]. Notably, cigarette compounds also diminish intracellular antioxidant mechanisms such as superoxide dismutase (SOD) and glutathione peroxidase (GPx) [11]. This oxidative protective impairment further contributes to increased inflammation and mitochondrial dysfunction.

In this context, the risk of CVD in smoking pre-menopausal females is often underestimated, due to the assumption that females are more protected against CVD development than males [12]. This is emphasized by the fact that endogenous-estrogen, 17β-estradiol (E2), the most potent human estrogen, affects almost every tissue or organ system, including the heart and blood vessels where it is linked in premenopausal women to NO production, blood pressure lowering actions, and multiple cardiovascular protective effects [13]. The latter are characterized in general by preserved cardiac function, decreased ROS generation, decreased apoptosis, decreased fibrosis and collagen deposition, and decreased immune cell infiltration and inflammatory cytokine generation [13]. However, research has shown that CS may interfere with the body's ability to utilize endogenous and exogenous estrogen [14]. Seemingly, smoking and estrogen have opposing effects on many common targets in the body. To date, data on the effects of chronic cigarette smoking on the heart in relation to ethinylestradiol (EE) oral contraceptive (OC) usage in premenopausal women is scarce. Therefore, we sought to investigate, for the first time, the mechanism of smoke cardiomyopathy in females using EE OCs.

## Material and methods

### Animals

This study was approved by the Institutional Animal Care and Use Committee (IACUC) of the American University of Beirut (AUB). Five-month old WT C57BL/J6 fertile female and male mice (25–30 g) were purchased from Charles River Laboratories (Wilmington, MA, USA) and housed under pathogen-free conditions with constant temperature and humidity control at the AUB animal care facility. Mice were provided sterile bedding and ad libitum access to water and rodent chow.

### Study timeline and experimental design

**Fig. 1:**
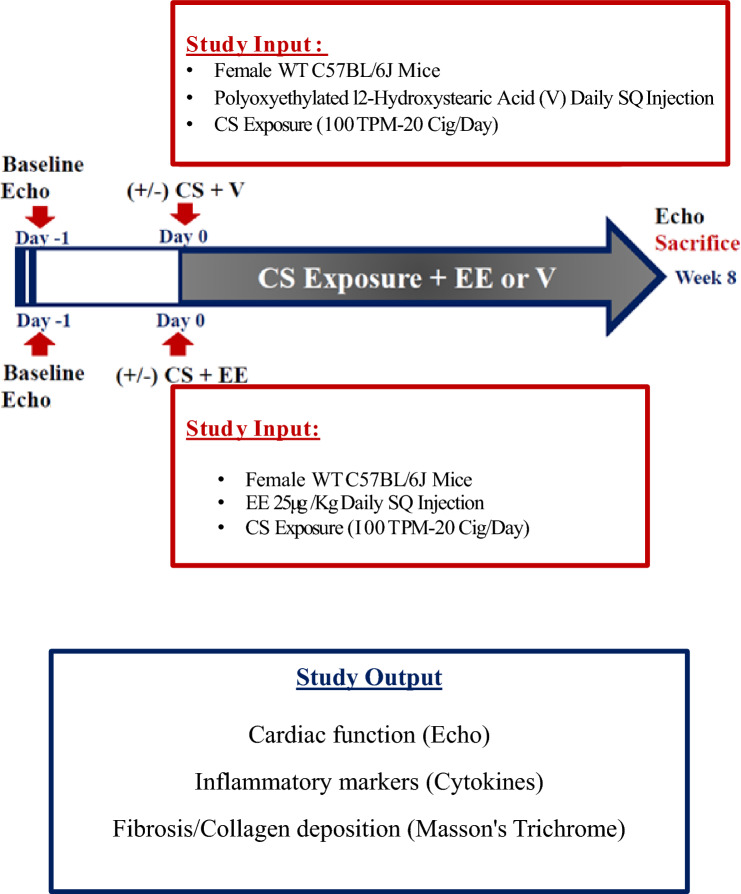
Study timeline. EE, ethinylestradiol; V, vehicle; CS, chronic cigarette smoking; WT, wild type; SQ, subcutaneous; TPM, total particulate matter; Cig, cigarette; Echo, echocardiography

The experimental design of this study is shown in Fig. 1. Baseline echocardiography (echo) was performed. Mice were then enrolled into 8 weeks of smoking exposure in parallel with subcutaneous (SQ) injections of EE or vehicle (V), with echo recorded every other week. Mice were sacrificed at week 8 and the organs were collected. Ovariectomy was performed as previously described [15], 3 weeks before being used in experiments.

### Echocardiography

Echocardiography was performed using the VEVO 2100 (FUJIFILM VisualSonics, Inc., Toronto, ON, Canada) with a frequency of 15.0 MHz. For imaging, animals were anaesthetized with isoflurane 2 to 3% in an oxygen mix chamber and placed on an electrically heated platform in a supine position. Body temperatures (maintained at 37 °C), heart rates and respiratory rates were continuously monitored throughout the procedure via an Indus Mouse-Monitor Heated Surgical Platform and the depth of anaesthesia was adjusted to maintain heart rate at 400–500 beats per minute. M-mode and B-mode echocardiography images of the left ventricle (LV) were obtained in the parasternal long-axis and short-axis views. Measurements at baseline and at 8 weeks right before sacrifice were acquired. For all animals three to four beats were measured using the same transducer position and mean calculations were obtained from three consecutive cardiac cycles. Fractional shortening (FS), ejection fraction (EF), left ventricular end‐systolic diameter (LVESD), left ventricular end‐diastolic diameter (LVEDD), left ventricular end‐systolic volume (LVESV) and left ventricular end‐diastolic volume (LVEDV) were recorded.

### Chronic smoking exposure

Upon arrival to the smoking exposure room and following a short acclimatization period, female mice were exposed to cigarette smoke (CS) using an exposure apparatus (ONARES, CH Technologies, USA). This apparatus includes a smoke generator with a mixing/conditioning chamber and a “nose only” rodent exposure carousel. It allows for exposure to mainstream smoke from a cigarette in conscious, restrained rodents. This system has been extensively used to study smoking-related diseases. Mice in the smoking groups (V + CS and EE + CS) received CS twice daily (7 days/week) for 8 weeks. Cigarettes were placed into the cigarette puffer, and a peristaltic pump was used to generate puffs at a frequency of 1 puff/minute, duration of 2.5 s, and a volume of 5 ml per puff generated from 3R4F cigarettes (University of Kentucky, Lexington, KY, USA). 3R4F are scientifically prepared cigarettes concentrated with toxins and chemicals, making the study timeline suitable to observe the effects of smoking on mice. Animals received two 60 min CS sessions per day allowing a total particular matter (TPM) concentration of about 100g/cm^3^/mouse/session (100 mg TPM, 9.4 mg tar, and 0.726 mg nicotine per cigarette).

### Drug delivery

Stock solutions of EE (Sigma-Aldrich, St. Louis, MO) for injections were dissolved in a vehicle (V) of 0.1% solution of polyoxyethylated 12-hydroxystearic acid (Sigma-Aldrich) and additional dilutions were made using normal saline. Mice were subcutaneously injected with 25 μg/kg/day EE, daily for 21 days (3 weeks) per cycle, for a total of 2 cycles in 8 weeks. Vehicle was given to control groups (100 μL/day). EE contraceptives are widely adapted in rodent research and are used in this study at a human-equivalent dose. The EE dosage was selected based on previous reports and also to be comparable to clinically relevant levels during oral contraceptive therapy daily regimen that an average 60–70 kg woman would be prescribed in an oral contraceptive [15].

### Necropsy

At the end of the study, all mice were sacrificed. Mice were anesthetized with 3% isoflurane vapor (Forane®) diluted with O_2_. They were then injected with heparin for 5 min prior to sacrifice to facilitate blood collection. After that, plasma was separated and mixed with protease inhibitors and snap-frozen in liquid nitrogen for further analysis. Hearts were isolated from all mice, the right ventricle was removed, and the rest was divided into 2 sections: one section was immediately suspended in formalin-containing storage vials for histology analysis and the other was snap-frozen in liquid nitrogen and stored at − 80 °C for molecular analysis.

### Histology

After formalin (10%) fixation for 2 days in a biopsy cassette and at room temperature, left ventricular transactional cuts were dehydrated and embedded in paraffin. Tissue sections from paraffin blocks (4 μm thickness) were mounted on glass slides for further different staining.

### Hematoxylin and Eosin (H & E) staining

Briefly, heart mid sections of 5 μm thickness were taken for each mouse, de-paraffinized, rehydrated, and stained with H & E stain for 10 min. The slides were then washed and examined under a light microscope. Cross-sectional area of 100 cells of each of the 4 groups: V, V + CS, EE, and EE + CS was measured using Image J software (https://imagej.nih.gov/ij/).

### Masson’s trichrome staining

In order to detect collagen deposition, tissue slides were stained with Masson’s Trichrome. Briefly, after dewaxing and hydration according to manufacturer’s protocol (Connective Tissue Stain, Abcam, ab150686) tissues were soaked in Boudin solution for 1 h at 56 °C, washed in running tap water, and then rinsed in distilled water. A second washing step was necessary after 10 min of incubation in hematoxylin, and then slides were stained in Biebrich scarlet acid fuchsin for 10 min. Sections were rinsed in water and incubated in phosphomolybdic phosphotungstic acid solution for 10 min. After that, they were transferred to aniline blue solution and stained for 5 min and finally mounted and observed using light microscopy. Fibrosis were measured using Image J software. For each section, ten fields were randomly imaged at 10 × magnification. Perivascular fibrosis was excluded from the analysis. The average measurement for all mice within the same group was then calculated.

### Western blotting

Snap-frozen left ventricular sections of the heart were ground in liquid nitrogen with a mortar and pestle and then homogenized in lysis buffer containing protease and phosphates inhibitors. This step was followed by centrifugation (12,000x*g* at 4 °C) for 10 min. Protein concentrations were quantified using a DCTM Protein Assay II kit (Bio-Rad, Hercules, CA, USA). Equal amounts of protein samples were loaded in wells of a 10 or 12% gel and run until the dye front reached the bottom of the gel. Separated proteins were transferred onto methanol-activated PVDF membranes at 4 °C for 1.5 h at 90 V. The membranes were blocked with 5% fat-free milk prepared in Tween PBS solution at room temperature for 1h and incubated with primary antibodies and glyceraldehyde 3-phosphate dehydrogenase (GAPDH); the latter was used to ensure equal loading of samples. Immunoblots were then probed with the appropriate secondary antibody. All antibodies are listed in Table 1. The immunoreactive bands were visualized with ECL chemiluminescence detection kit (Thermo Fisher Scientific). Image J software (https://imagej.nih.gov/ij/) was used to quantify the intensity of bands and to normalize GAPDH protein levels in each group to confirm any up or down-regulation of the targeted protein.

**Table 1:** List of antibodies

Antibody (dilution)	Catalogue number	Commercial source
Anti-IL13 (1/500–1/1000)	ab106732	abcam
Anti-IL4 (1/1000)	ab9728	abcam
Anti-IL1beta (1/500)	ab9722	abcam
Anti-IL13 (1/500–1/1000)	ab106732	abcam
Anti-IL10 (1/1000)	ab9969	abcam
Anti-PARP-1 (1/500–1/1000)	ab227244	abcam
Anti-GAPDH (1/500–1/1000)	ab8245	abcam
Secondary anti-mouse (1:10,000–1:200,000)	115-035-003	Jackson ImmunoResearch
Secondary anti-rabbit (1:10,000)	ab8245	abcam

### Reverse transcription-quantitative polymerase chain reaction (RT-qPCR)

Snap-frozen heart tissues were used for RNA extraction. Briefly, mid-sections from the left ventricle were grounded in liquid nitrogen by a mortar and pestle then total RNA was isolated using TRIzol according to manufacturer’s instructions (Thermo Fisher Scientific, Grand Island, NY, USA). NanoDrop® ND-1000 UV–Vis spectrophotometer was used for RNA quantification. The purity of extracted RNA was assessed using the absorbance ratio of 260 to 280 nm, where a value of 1.8–2.0 indicates good-quality of RNA. To remove contaminating DNA, RNAs were treated with deoxyribonuclease I (Thermo, USA). qPCR was used to quantify differences in mRNA expression. Gene expressions were monitored using SYBR® Green PCR Master Mix (Bio-Rad, Hercules, CA, USA). GAPDH expression was used to normalize gene expressions between different samples. cDNA was synthesized from RNA using Revert Aid 1st Strand cDNA synthesis kit (Thermo, USA), followed by real-time PCR analysis in a CFX96 real-time PCR instrument (Bio-Rad, Germany). For the qPCR reaction, cDNA was loaded in duplicates of each of the forward and reverse primers of the gene of interest and mixed with SYBR Green. Negative control (RNAs free water) was used to check for nonspecific amplification. Fold expressions were normalized relative to the control and were calculated and plotted using Bio-Rad CFX Manager to compare differential gene expressions. The sequences of primers are listed in Table 2.

**Table 2:** List of primers

Primers	Sequences	
	Forward	Reverse
IL-1-β	5′-TGG TGT GTG ACG TTC CCA TT-3′	5′-TGT CGT TGC TTG GTT CTC CT-3′
IL-6	GAACAACGATGATGCACTTGC	5′-TCCAGGTAGCTATGGTACTCC-3′
IL-13	5′-TGTGTCTCTCCCTCTGACCC-3′	5′-CAGGGCTACACAGAACCCG-3′
TNF-α	5′-AATGGGCTCCCTCTCATCAGTTC-3′	5′-TCTGCTTGGTGGTTTGCTACGAC-3′
NOX-4	5′-ACCAAATGTTGGGCGATTGTG-3′	5′-GGCTACATGCACACCTGAGA-3′
SOD-1	5′-TGTTGGAGACCTGGGCAATG-3′	5′-ACGGCCAATGATGGAATGCT-3′
GAPDH	5′-GGGGCTCTCTGCTCCTCCCTG-3′	5′-CGGCCAAATCCGTTCACACCG-3′

*IL-1* interleukin 1, *IL-6* interleukin 6, *IL-13* interleukin 13, *TNF-α* tumor necrosis factor alpha, *SOD-1* superoxide dismutase 1, *NOX-4* NADPH oxidase 4, *GAPDH* glyceraldehyde 3-phosphate dehydrogenase

### Statistical analysis

Data are shown as mean ± SEM. Statistical analyses were performed using GraphPad Prism 9 (GraphPad Software, San Diego, CA; https://www.graphpad.com). A difference of *P* < 0.05 was considered significant. The data for female mice was analyzed using 2-way ANOVA, while the data for male mice was analyzed using unpaired t-test. Prior to analysis, normality tests including the D'Agostino-Pearson omnibus, Anderson–Darling, Shapiro–Wilk, and Kolmogorov–Smirnov test were applied to ensure data distribution met assumptions for parametric testing. With all datasets passing at least one normality test, validation for the application of a 2-way ANOVA was achieved. In case of significant interaction or if one or both main effects was significant, a Tukey post-test was performed. When representative images are shown, the selected images were those that most accurately represented the average data obtained in all the samples.

## Results

### The EE + CS combination resulted in adverse structural and functional cardiac remodeling in premenopausal female mice

**Fig. 2:**
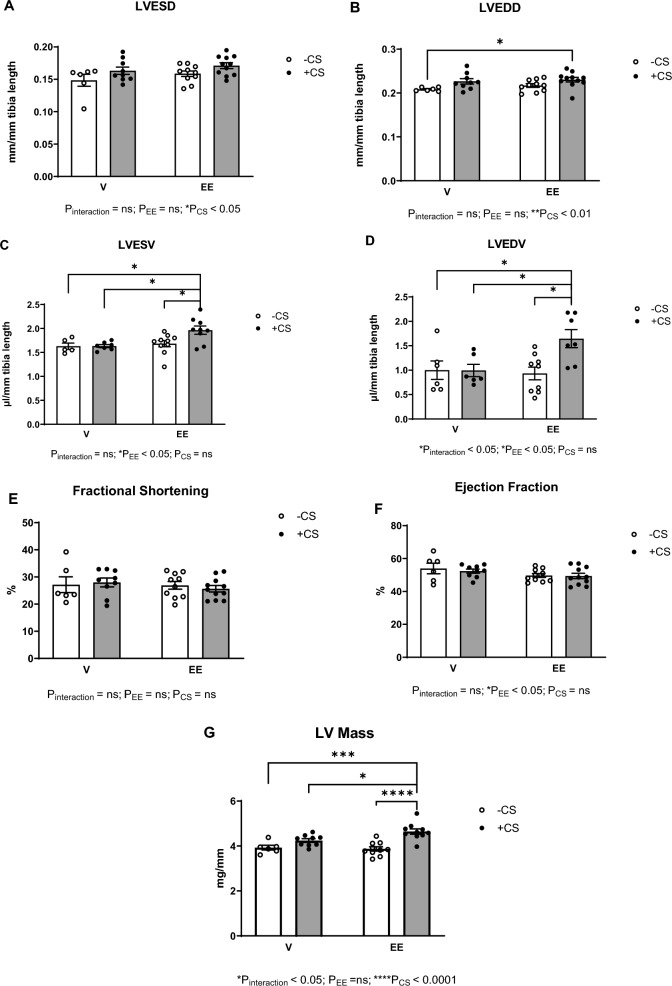
Effects of EE + CS on cardiac function and structure. The combination of EE and CS did not alter (**A**) LVESD but increased (**B**) LVEDD, (**C**) LVESV, and (**D**) LVEDV. The combination of EE and CS did not affect (**E**) fractional shortening and (**F**) ejection fraction but increased (**G**) LV mass. Values are normalized to tibia length. CS, chronic smoking; EE, Ethinylestradiol; V, vehicle; LVESD, left ventricular end-systolic diameter; LVEDD, left ventricular end-diastolic diameter; LVESV, left ventricular end-systolic volume; LVEDV, left ventricular end-diastolic volume. Data were analysed for significance using two-way ANOVA. All bars represent mean ± SEM (^*^P ≤ 0.05; ^**^P ≤ 0.01; ^***^P ≤ 0.001; *n* = 5–11)

CS increased both LVESD and LVEDD, but no effect of EE was observed (Fig. 2A, B). A modest increase in LVESV with EE was in evidence in mice exposed to CS (Fig. 2C); however, the combination of EE and CS markedly increased LVEDV (Fig. 2D). The fractional shortening and ejection fraction were similar across all groups (Fig. 2E, F). However, CS enhanced LV mass, with the EE + CS group showing a significant increase in LV mass compared to all other groups (Fig. 2G).

**Fig. 3:**
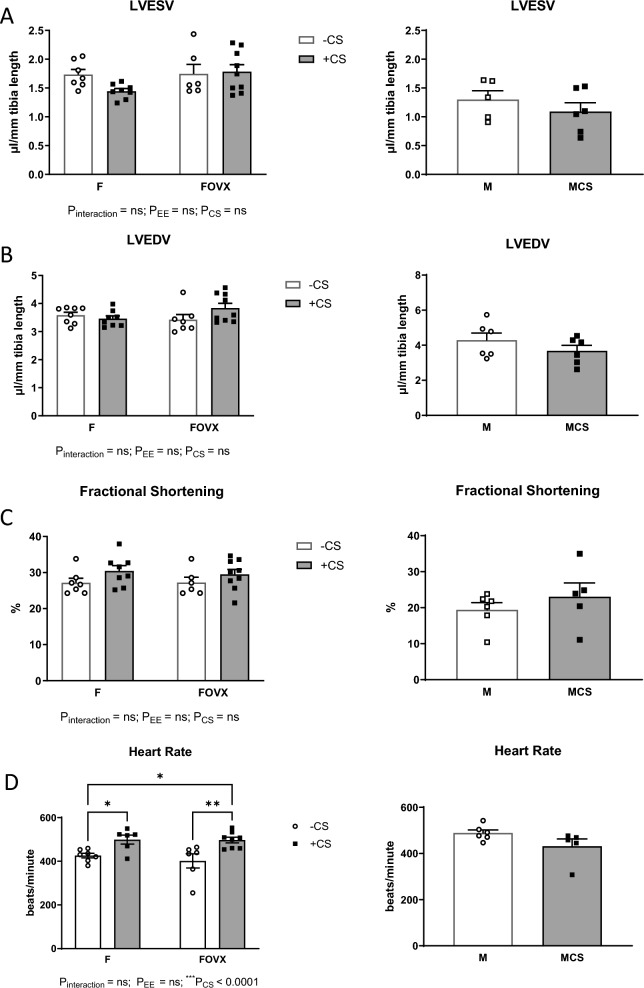
The effect of CS on cardiac performance in ovariectomized females and male mice. Smoking did not have an impact on (**A**) LVESV, (**B**) LVEDV, and (**C**) fractional shortening in either the male or the female mice, regardless of ovariectomy. Smoking stimulated the (**D**) heart rate of female mice only. CS, chronic smoking; F, females; FOVX, ovariectomized females; M, males; MCS, smoking males; LVESV, Left ventricular end-systolic volume; LVEV, left ventricular end-diastolic volume. Two-way ANOVA was used to analyze the data of the female mice while unpaired t-test was used to analyze the data of the male mice. All bars represent mean ± SEM. (^***^P ≤ 0.001; n = 6–9 for females; n = 5–6 for males)

To investigate the effects of sex, echocardiographic measurements were conducted on male and female mice. Additionally, to examine whether endogenous estrogen and exogenous estrogen have differential effects in smoking females, measurements were performed using ovariectomized (OVX) females. Our findings did not reveal any noticeable changes in the left ventricle end-systolic and end-diastolic volumes, as well as the fractional shortening, in both male and female smokers (Fig. 3A–C). However, unlike male mice, smoking increased the heart rate of female mice irrespective of estrogen presence (Fig. 3D).

### The EE + CS combination resulted in upregulated expression of inflammatory marker mRNAs, but not TNFα gene expression and serum levels

**Fig. 4:**
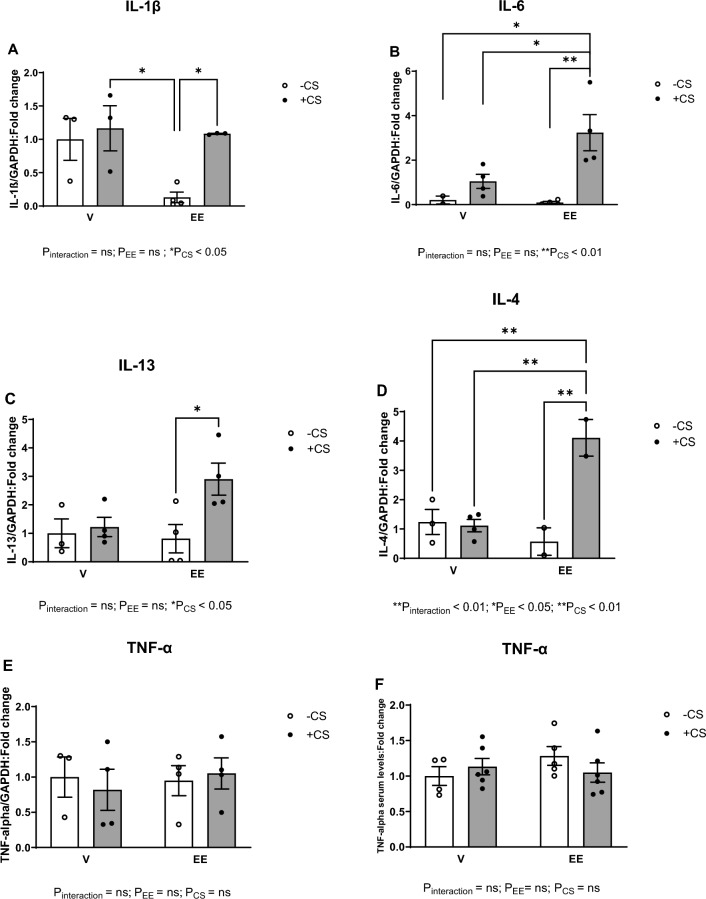
Effects of EE + CS on gene expressions of proinflammatory markers. The combination of EE and CS increased the mRNA expression levels of (**A**) IL-1β, (**B**) IL-6, (**C**) IL-13, and (**D**) IL-4. However, the combination did not alter the (**E**) mRNA expression and (**F**) serum levels of TNF-α. CS, chronic smoking; EE, Ethinylestradiol; V, vehicle; LV, left ventricular. Data were analysed for significance using two-way ANOVA. All bars represent mean ± SEM (^*^P ≤ 0.05; ^**^P ≤ 0.01; n = 2–6)

Unlike the V-treated groups that showed comparable levels of IL-1β and IL-6 mRNA expressions in the absence and presence of CS, both IL-1β and Il-6 pro-inflammatory gene expression increased in the left ventricles of EE + CS exposed mice after 8 weeks, compared to the EE-CS group (Fig. 4A, B). Moreover, IL-6 gene expression was significantly increased in the EE + CS group compared to the vehicle-treated groups (Fig. 4B). The EE + CS group also showed a significant increase in IL-13 gene expression compared to the EE-CS group (Fig. 4C). IL-4 gene expression showed a significant effect of the EE + CS combination compared to all other conditions (Fig. 4D). On the other hand, EE administration or CS exposure did not affect TNFα gene expression (Fig. 4E) or serum levels (Fig. 4F) alone or in combination.

### The EE + CS combination resulted in a significant upregulations of inflammatory protein expression and cell-death markers

**Fig. 5:**
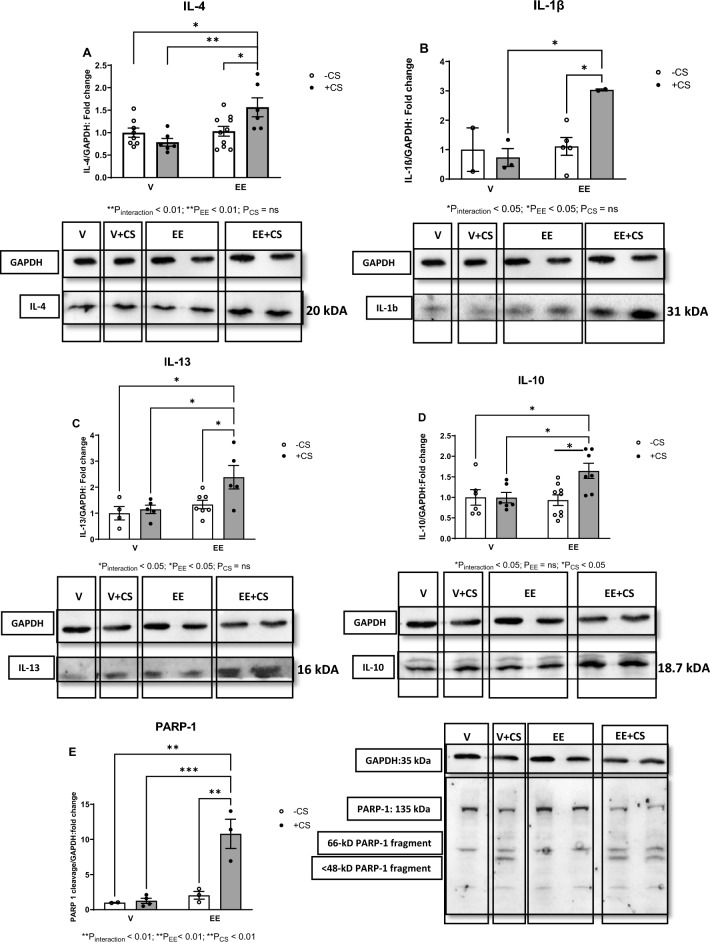
Effects of EE + CS on protein expressions of proinflammatory markers. The combination of EE and CS increased the protein expression levels of (**A**) IL-4, (**B**) IL-1β, (**C**) IL-13, (**D**) IL-10, and the apoptotic biomarker (**E**) PARP-1. CS, chronic smoking; EE, Ethinylestradiol; V, vehicle; LV, left ventricular; IL, interleukin; GAPDH, glyceraldehyde 3-phosphate dehydrogenase; TNF-α, tumor necrosis factor alpha; PARP-1, poly (ADP-ribose) polymerase 1. Data were analysed for significance using two-way ANOVA. All bars represent fold change ± SEM after normalization to GAPDH (^*^P ≤ 0.05; ^**^P ≤ 0.01; ^***^P ≤ 0.001; n = 2–10)

In agreement with the PCR results, we found a significant increase in the protein expression levels of IL-4, IL-1β, IL-13, IL-10 inflammatory markers in the EE + CS group (Fig. 5A–D). Likewise, the same group presented with a significant increase in PARP-1 protein expression, as a marker of increased cellular stress and cell programmed death, in comparison to all of the other groups (Fig. 5E).

### The EE + CS combination did not affect the mRNA expression levels of NOX-4 and SOD-1

**Fig. 6:**
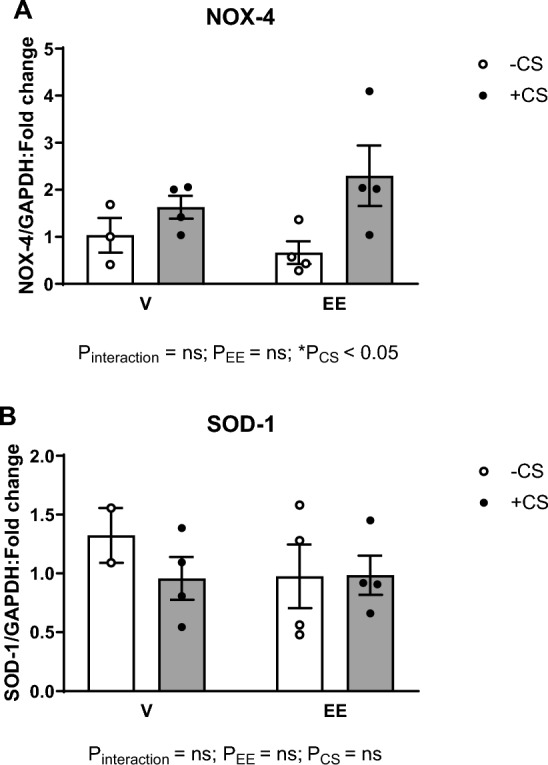
Effects of EE + CS on gene expressions of oxidative stress markers. The combination of EE and CS did not affect the mRNA expression levels of (**A**) NOX-4 and (**B**) SOD-1. CS, chronic smoking; EE, Ethinylestradiol; V, vehicle; LV, left ventricular; NOX-4, NADPH oxidase 4; SOD, superoxide dismutase; GAPDH, glyceraldehyde 3-phosphate dehydrogenase. Data were analysed for significance using two-way ANOVA. All bars represent fold change ± SEM (*P = 0.05; n = 2–3)

The EE + CS combination tended to increase the expression of the NOX-4 gene compared to the EE-CS group (Fig. 6A), although this did not reach significance. Moreover, no change was observed in SOD-1 expression among all groups (Fig. 6B).

### The EE + CS combination resulted in collagen deposition in the left ventricular cardiac tissue but did not affect the cardiomyocyte cross-sectional area

**Fig. 7:**
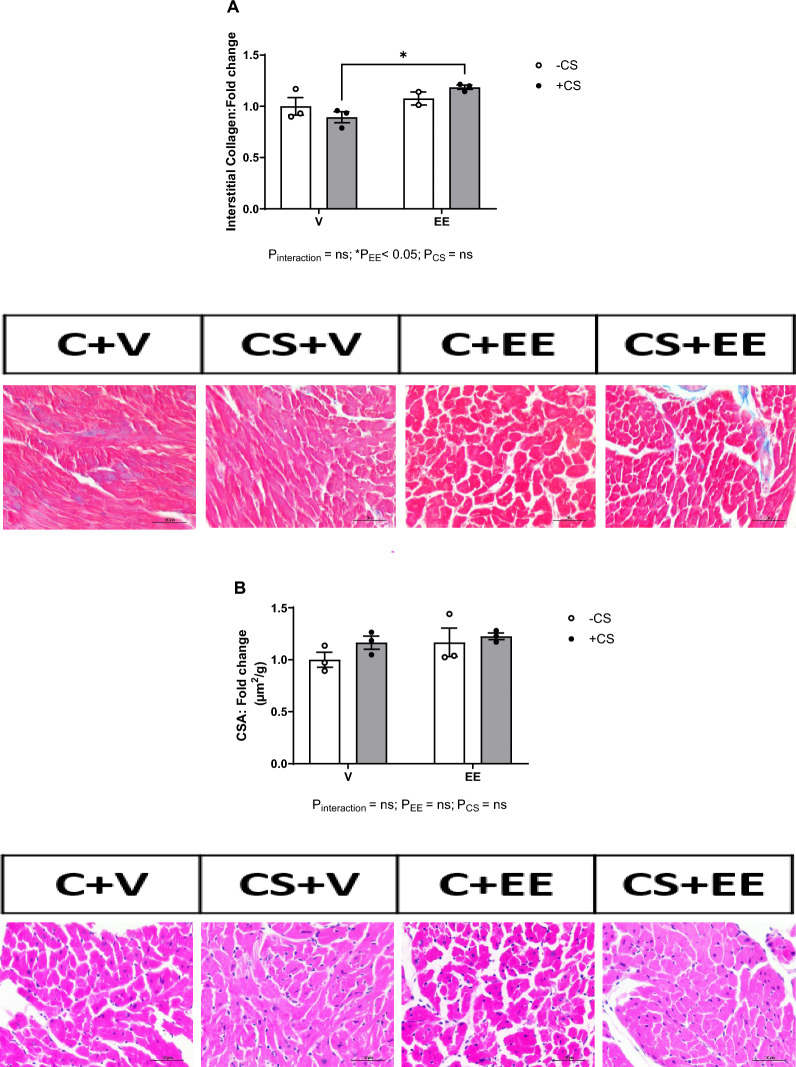
Effects of EE + CS on cardiac collagen and myocyte size. The combination of EE and CS increased (**A**) cardiac interstitial fibrosis but did not affect (**B**) cardiomyocytes cross-sectional area. CS, chronic smoking; EE, Ethinylestradiol; V, vehicle. Data were analysed for significance using two-way ANOVA. All bars represent fold change ± SEM (*P < 0.05; n = 2–3). Scale bars = 100 µm

LV cardiac tissue from the EE + CS group showed significantly increased deposition of interstitial collagen compared to the V + CS group (Fig. 7A). Importantly, EE is the primary factor contributing to this variation, rather than CS. The administration of EE resulted in no change in the cross-sectional area in the presence or the absence of CS (Fig. 7B) indicating that there was no sign of cardiac hypertrophy at the histological level.

## Discussion

In this study, we assessed whether the effects of estrogen on the heart were influenced by cigarette smoking in female mice. We observed that in response to cigarette smoking exposure, estrogen administration caused an increase in diastolic LV volume and an increase in LV mass. In addition, increases were observed in cardiac IL-4, IL-1β, IL-13, IL-10, and PARP-1 expression indicating an active inflammatory response. Greater interstitial collagen was also seen in cigarette smoke-exposed mice treated with estrogen. These observations are consistent with early stages of adverse cardiac remodeling as seen for instance with dilated cardiomyopathy [16]. These findings have relevance to understanding the risk of CVD due to smoking in premenopausal females, especially in the context of oral contraceptive usage.

Estrogen receptors are expressed by cardiomyocytes and orchestrate a myriad of genomic and non-genomic effects on the heart [17]. Cardiovascular function is enhanced by endogenous estrogen; however, research has shown that CS may interfere with the body's ability to utilize endogenous and exogenous estrogen [14]. Some studies suggest that cigarette smoking is more harmful in young (premenopausal) women than in men, which may be due to CS-induced changes in estrogen activity [18]. It has been demonstrated that CS has anti-estrogenic effects due to changes in estrogen metabolism [19, 20], by increasing the 2-hydroxylation stage of estradiol metabolism and thereby reducing estrogen availability and activity [21, 22]. Tobacco smoke components lead to the upregulation of a number of genes, including members of the cytochrome P450 (CYP) family, in particular CYP1B1 and CYP1A; the latter and CYP1A2 are the primary enzymes in humans for catalyzing the 2-hydroxylation of estradiol [23]. Another study found that when taking oral estrogen replacement therapy (ERT), CS reduced estrogen levels by 40–70%, but when estrogen was applied transdermally at a low dosage to bypass liver metabolism smoking only caused a small change in estrogen levels [24]. These findings can be attributed to CS increasing the hepatic metabolism of oral estrogens, thus preventing the body from using drug-derived estrogens by converting them to forms that are less active. In addition, the 2-hydroxylation of estradiol results in the production of metabolites that have been associated with endometriosis and pain [25].

Our study findings indicate that, at this stage, estrogen does not appear to influence the functional responses of female mice hearts to smoking. We observed no sex differences in this respect. However, we did find that smoking increased the heart rate in female mice, regardless of whether they had undergone ovariectomy. This suggests that females may be more attuned to smoking, potentially elevating the risk of cardiac function deterioration over time. Cardiac performance in male mice was not affected by CS during the timeframe studied. However, we and others have previously reported that cigarette smoking results in a relatively modest increase in male mice in both LVEDD and LVESD, suggesting that timing or other factors are involved [26, 27].

On the other hand, when female mice were administered exogenous estrogen while also being exposed to smoking, they experienced ventricular dilation, increased left ventricular mass, and cardiac fibrosis. This demonstrates the negative effects of this combination on cardiac homeostasis. At the molecular level, exposure to smoking and exogenous ethinylestradiol administration led to increased expression of cytokines linked to both type 1 and type 2 inflammatory responses. Numerous studies have reported that cigarette smoking is strongly associated with the development of a systemic inflammatory response and increased risk of atherosclerotic cardiovascular disease [28]. CS has direct adverse effects on the myocardium, resulting in what is referred to as smoking cardiomyopathy [6]. Our findings indicate that estrogen supplementation may exacerbate smoking cardiomyopathy and the risk of cardiovascular disease. IL-4 and IL-13 are associated with cardiac fibrosis and hypertrophy [29–31], while IL-1β mediates inflammation, endothelial dysfunction and myocardial injury [32]. IL-10, traditionally considered anti-inflammatory, can paradoxically exacerbate cardiac remodeling under certain conditions [29]. PARP-1, involved in DNA repair and inflammation, also contributes to the pathophysiology of CVDs [33]. The complex roles of these inflammatory markers in cardiac remodeling underscore the intricate interplay between chronic smoking, estrogen supplementation and cardiovascular health. Further mechanistic studies are needed to elucidate the specific pathways by which these cytokines contribute to adverse cardiac remodeling in the context of estrogen supplementation and smoking.

Our study has a few limitations. For one thing, we did not measure 2-hydroxylation of estradiol or blood estrogen levels to interpret whether endogenous and exogenous estrogen are differentially regulated. Nor did we examine immune cell infiltration into the heart. In addition, we did not look at what effect estrogen treatment may have had on the detrimental effects of cigarette smoking on vascular histology and function. In addition, with regard to OCs, estrogen and progesterone combination are commonly used as progesterone helps to regulate some effects of estrogen. This combination may have different results from what we found and needs future study. We also did not look at the effects of EE on males, which is relevant to defining the cardiovascular risks of CS to transgender females. In conclusion, the findings of our study indicate that in combination with cigarette smoking estrogen has harmful effects on the hearts of fertile female mice that are consistent with adverse cardiac remodeling. Additional studies are warranted to decipher the molecular basis for those actions.

## Data Availability

No datasets were generated or analysed during the current study.

## Abbreviations

CAD: Coronary artery disease
CHD: Coronary heart disease
CS: Chronic smoking or cigarette smoke
CVD: Cardiovascular disease
E2: 17β-Estradiol
ECL: Enhanced chemiluminescence
EE: Ethinylestradiol
EF: Ejection fraction
ERT: Estrogen replacement therapy
FS: Fractional shortening
GAPDH: Glyceraldehyde 3-phosphate dehydrogenase
GPx: Glutathione peroxidase
IL: Interleukin
LV: Left ventricle/ventricular
LVEDD: Left ventricular end‐diastolic diameter
LVEDV: Left ventricular end‐diastolic volume
LVESD: Left ventricular end‐systolic diameter
LVESV: Left ventricular end‐systolic volume
NO: Nitric oxide
NOX-4: NADPH oxidase 4
OC: Oral contraceptive
OVX: Ovariectomized
PARP-1: Poly [ADP-ribose] polymerase 1
ROS: Reactive oxygen species
SOD: Superoxide dismutase
TPM: Total particular matter
